# When Cognitive Decline and Depression Coexist in the Elderly: CSF Biomarkers Analysis Can Differentiate Alzheimer's Disease from Late-Life Depression

**DOI:** 10.3389/fnagi.2018.00038

**Published:** 2018-02-23

**Authors:** Claudio Liguori, Mariangela Pierantozzi, Agostino Chiaravalloti, Giulia M. Sancesario, Nicola B. Mercuri, Flaminia Franchini, Orazio Schillaci, Giuseppe Sancesario

**Affiliations:** ^1^Neurophysiopathology Unit, Department of Systems Medicine, University of Rome Tor Vergata, Rome, Italy; ^2^Neurology Unit, Department of Systems Medicine, University of Rome Tor Vergata, Rome, Italy; ^3^Department of Biomedicine and Prevention, University of Rome Tor Vergata, Rome, Italy; ^4^IRCSS Neuromed, Pozzilli, Italy; ^5^IRCCS Fondazione Santa Lucia, Rome, Italy

**Keywords:** late-life depression, Alzeimer's disease, CSF biomarkers, beta-amyloid 1-42, tau proteins, 18FFDG PET scan, MMSE, PHQ-9

## Abstract

Late-life depression (LLD) and Alzheimer's Disease (AD) are the two most frequent neuropsychiatric disorders affecting elderly. LLD and AD may clinically present with depressive and cognitive symptoms. Therefore, when cognitive decline is coupled with depression in the elderly, the differential diagnosis between LLD and AD could be challenging. The aim of the present study was to evaluate in a population of elderly patients affected by depression and dementia the usefulness of CSF AD biomarkers (tau proteins and β-amyloid_42_–Aβ_42_) and 2-[18F]fluoro-2-deoxy-d-glucose positron emission tomography (18FFDG-PET) in early differentiating LLD from AD. Two hundred and fifty-six depressed and demented patients, after performing CSF AD biomarkers and 18FFDG-PET, were distributed in two groups on the basis of the current diagnostic guidelines for AD (*n* = 201) and LLD (*n* = 55). Patients were then observed for 2 years to verify the early diagnosis. After the 2 year follow-up we compared AD and LLD patients' CSF and 18FFDG-PET data obtained at baseline to a group of age- and sex-matched controls. We found CSF Aβ_42_ levels significantly higher in LLD compared to AD patients. Remarkably, CSF Aβ_42_ levels of LLD patients (range between 550 and 1204 pg/mL) did not overlap with those of AD patients (range between 82 and 528 pg/mL). Moreover, we documented no differences in CSF AD biomarkers (Aβ_42_ and tau proteins) when comparing LLD patients to controls. In addition, AD patients showed the significant reduction of 18FFDG-PET uptake in temporo-parietal regions compared to both controls and LLD. Conversely, LLD and control groups did not differ at 18FFDG-PET analysis, although LLD patients showed heterogeneous patterns of glucose hypometabolism involving cortical and subcortical brain areas. It is noteworthy that at the end of the clinical follow-up, patients owing to AD group showed the expected significant decline of cognitive performances, whereas patients assigned to LLD group improved cognition as depressive symptoms recovered. Hence, in case of co-existence of cognitive impairment and depression in the elderly, we propose CSF AD biomarkers analysis to early differentiate LLD from AD and properly target the patient's therapeutic strategy and clinical follow-up.

## Introduction

Alzheimer's Disease (AD) is the most common progressive form of neurodegenerative dementia in the older population (McKhann et al., 2011). Neuropathologically, AD is marked by extracellular amyloid plaques, composed of beta-amyloid_1−42_ (Aβ_42_) peptides, intracellular neurofibrillary tangles (NFTs), containing tau proteins, and both synaptic and neuronal loss (Yaari and Corey-Bloom, 2007). Hence, Aβ_42_, total tau (t-tau) and phosphorylated-tau (p-tau) proteins become the established cerebrospinal fluid (CSF) biomarkers to support AD diagnosis (Kester et al., 2009).

As largely documented, AD is primarily characterized by cognitive decline, but may often present concomitant with depression, which could even precede the neurodegenerative process and the clinical signs of dementia (Fischer, 1996; Kobayashi and Kato, 2011; McKhann et al., 2011). Latelife depression (LLD) recently emerged as a definite neuro-psychiatic disorder with an increasing prevalence in the elderly (American Psychiatric Association, 2000). Since LLD may cause mental deterioration with reduced initiative, memory complaints and attention deficits, it actually represents an healthcare problem (American Psychiatric Association, 2000; Naismith et al., 2012; Richard et al., 2013). Therefore, both AD and LLD significantly affect quality of life of patients and caregivers, since clinical symptoms often impair activities of daily living (Fischer, 1996; Lyketsos et al., 2002; Naismith et al., 2012; Forlani et al., 2014; Morimoto et al., 2015).

Depression in older adults, often identified as LLD, appears to differ from depression earlier in life, with increased heterogeneity across the adult population. LLD has been associated with cognitive impaitment with predominant involvement of executive dysfunction and memory loss (Alexopoulos et al., 2005). LLD has been consequently studied as a early stage of AD and CSF biomarkers have been evaluated in patients affected by LLD. Accordingly, those studies evaluated cognitive intact patients affected by LLD in order to test if this condition may be considered a preclinical stage of AD pathology. However, very recent studies did not document pathological cerebral beta-amyloid pathology and/or hippocampal volume reduction in LLD patients (De Winter et al., 2017; Li et al., 2017). Therefore, it is actually under debate if LLD has a relationship with brain amyloid pathology and thus AD.

On the basis of these observations, elderly can be potentially affected by two different clinical patterns of mixed affective-cognitive deficits: (i) cognitive impairment due to AD with concomitant depressive symptoms; (ii) LLD with associated cognitive deficits mimicking dementia.

Considering the high prevalence of both AD and LLD in the elderly, establishing which pathological process underlies the clinical picture characterized by cognitive impairment coupled with depression can represent a challenging issue. The clinical follow-up nowadays represents the best approach to confirm the differential diagnosis between AD and LLD in patients affected by both depression and dementia. Accordingly, since the different mechanisms at the basis of AD and LLD, the early differential diagnosis between these disorders is an important starting point for choosing the most appropriate patients' treatment. At this aim, in this study we studied elderly patients affected by dementia coupled with depression undergoing a complete diagnostic work-up, including 2-[18F]fluoro-2-deoxy-d-glucose positron emission tomography (18FFDG-PET) and lumbar puncture (LP) for CSF biomarkers analysis (tau proteins and β-amyloid_42_–Aβ_42_), and then followed for 2 years. Once the clinical follow-up confirmed the diagnosis of AD and LLD, we tested the effectiveness of CSF AD biomarkers and 18FFDG-PET obtained at baseline in early differentiating LLD from AD.

## Methods

### Participants and study procedures

In our study we observed a population of untreated elderly patients (≥65 year old) affected by cognitive impairment and depression, who were admitted to the Neurological Clinic of University of Rome “Tor Vergata” between January 2010 and December 2014. All patients were affected by cognitive decline with insidious onset, progressive worsening of cognition, and significant alteration of memory performances in the previous months. Moreover, patients showed depressive symptoms started in the older age. Therefore, patients to be included in this observation had to show a Mini Mental State Examination (MMSE) score ranging between 15 and 24 and a Patient Health Questionnaire 9 (PHQ-9) score ≥10 (Löwe et al., 2004). PHQ-9 is a self-report questionnaire, commonly used to screen for depression, consisting of nine questions asking about depression symptoms with a recommended cut-off score of 10 (Manea et al., 2012). PHQ-9 can be also used to confirm the diagnosis of a major depressive episode and to monitor the severity of depressive symptoms, also assessing response to treatments (Löwe et al., 2004; Manea et al., 2012).

At baseline all patients underwent history, neurologic examination, blood sample, electroencephalogram (EEG), brain magnetic resonance imaging (MRI), 18FFDG-PET, and LP for CSF biomarkers analysis (tau proteins and Aβ_42_). Once diagnostic work-up was completed, we excluded patients showing behavioral and cognitive symptoms suggestive of Frontotemporal Lobar Degeneration (FTLD), (Rascovsky et al., 2011) parkinsonisms, subclinical seizure at EEG, brain mass, and hydrocephalus at brain MRI. Then, we distributed the remaining patients in AD or LLD groups on the basis of the current diagnostic guidelines for AD and LLD (Yaari and Corey-Bloom, 2007; McKhann et al., 2011). The diagnosis of AD was established based on the recently proposed biomarkers diagnostic criteria for AD, which imply decreased CSF levels of Aβ_42_ associated to medial temporal lobe atrophy on MRI, cortical temporo-parietal hypometabolism on 18FFDG-PET and increased CSF levels of total-tau (t-tau) and phosphorylated-tau (p-tau) (McKhann et al., 2011). Patients included in the LLD group have to meet the following DSM-IV criteria for major depressive disorder: (1) depressed mood and/or a loss of interest/pleasure in daily activities for at least 2 weeks; (2) impaired social and/or occupational functioning as a result of depressive symptoms; (3) at least five of nine additional symptoms relating to appetite, sleep, etc (American Psychiatric Association, 2000).

Once the patients were distributed in the two groups, they started a 2-year clinical follow-up, counting MMSE to measure global cognitive status and PHQ-9 to assess depression. The psychologist (FF) performing the neuropsychological evaluations was blinded to the patients' clinical status and biomarkers analysis.

Considering that we performed an observational study, the patients were not assigned to a particular treatment during the follow-up, but treatments depended on patients' needs and current clinical practice. Therefore, chronic therapy with acetylcholinesterase inhibitors (AchEI), memantine, and antidepressants were administered in agreement with the current clinical practice. In particular, patients owing to the AD group started AchEI and/or memantine therapy according to dementia severity, and LLD patients started antidepressant treatments.

The study protocol was approved by the Ethical Committee of the University Hospital of Rome “Tor Vergata” and written informed consent was obtained from all participating in the study. The study was performed according to the STROBE statement.

### Control group

The control group included inpatients at the same clinic undergoing clinical neurologic examination, brain MRI, total body 18FFDG-PET, and LP for suspected malignancies, which were ruled out after diagnostic investigations. Inclusion criteria were clinical and instrumental data excluding central and peripheral nervous system diseases. Exclusion criteria were diabetes, depression or other psychiatric symptoms, cognitive decline, and CSF and brain 18FFDG-PET biomarkers suggestive of preclinical AD.

### CSF collection and analysis

All the CSF samples were obtained by LP performed in decubit position between 8:00 and 9:00 a.m. using an atraumatic needle. Blood specimens were also obtained at the same time of LP procedure. CSF samples were collected in polypropylene tubes using standard sterile techniques. The first 4 mL CSF sample was used for biochemistry routine analysis including total cell count. A second 4 mL CSF sample was centrifuged to eliminate cells and cellular debris and immediately frozen at −80°C until the analysis to assess t-tau, p-tau, and Aβ_42_ amount. The researcher (GMS), who performed the CSF analysis, was completely blinded to the patients' clinical status. Chemistry assays were carried out using commercially available kits following the manufacturer's specifications (Flex reagent cartridge, Dimension Vista System, Siemens Healthcare Diagnostics GmbH, Munich, Germany). The Aβ_42_, t-tau, and p-tau CSF levels were determined according to previously published standard procedures, using commercially available sandwich enzyme-linked immunosorbent assays (Innotest β-Amyloid 1-42, Innotest h-T-tau, InnotestPhospho-T-tau 181; Innogenetics, Ghent, Belgium) (Liguori et al., 2015). Aβ_42_, t-tau, and p-tau were dichotomized on the basis of previously established cut-off values: < 500 pg/mL for Aβ_42_, > 375 pg/mL for t-tau, and > 52 pg/mL for p-tau (Mulder et al., 2010; Duits et al., 2014; Liguori et al., 2016b, 2017) The t-tau/Aβ_42_ ratio was also calculated, and the cut-off > 0.52 was considered to represent the CSF AD profile (Liguori et al., 2016b).

### 18FFDG PET analysis

The PET/CT system Discovery VCT (GE Medical Systems, Tennessee, USA) was used to assess 18FFDG brain distribution in all patients and controls by means of a 3D-mode standard technique in a 256x256 matrix. Reconstruction was performed by a researcher (AC), who was blinded to patients' clinical status, as previously described (Liguori et al., 2016a).

### Data and statistical analysis

We used the Statistica 10.0 program (Statsoft Inc, Tulsa, OK, USA) for the statistical analysis. The Kolmogorov-Smirnov test was used to check for normal distribution of data. The one-way analysis of variance was used to compare demographical, clinical, and CSF data between more than two groups (AD vs. LLD vs. controls). The *post-hoc* analysis was performed using Tukey's honest significance test. MMSE and PHQ-9 data obtained at the time of diagnostic workup and at 2-year follow-up in LLD and AD groups were compared using paired *t-*test. *P*-value < 0.05 was considered to be statistically significant.

### 18FFDG-PET/CT image analyses

Differences in brain 18FFDG uptake were analyzed using statistical parametric mapping (SPM8, Wellcome Department of Cognitive Neurology, London, UK) implemented in Matlab 2012b (Mathworks, Natick, Massachusset, USA), as previously reported (Liguori et al., 2016a). The voxel-based comparisons were assessed for: (1) LLD vs. Controls; (2) LLD vs. AD. All the comparisons have been performed using a “two sample *t*-test” design model. In the obtained SPM maps, we searched for the brain areas with a significant correlation using a statistical threshold of *P* = 0.001, family wise error (FWE)-corrected for the problem of multiple comparisons, with an extent threshold of 100 voxels. Each 18FFDG-PET patient's image scan was tested for relative “hypometabolism” by comparison with the reference CG on a voxel-by-voxel basis using the general linear model, by means of the two sample *t*-test design of SPM8 as proposed previously by Perani (Perani et al., 2014). Age and sex was included as covariates in the SPM analyses. The measurements were assumed to be independent and have unequal variance between levels. Global normalization of voxel values used proportional scaling to a mean voxel value of 6.5 mg/100 mL/min to minimize inter-subject variability. Proportional scaling basically scales each image according to a reference count, which is the global brain activity to a physiologically realistic reference value of 6.5 mg/100 mL/min. The threshold was left at the default 0.8 value (i.e., the mean brain intensity was computed from only those voxels with intensity above 0.8 of the mean over the entire scan) (Perani et al., 2014). Voxel-wise comparisons were made using an explicit 18FFDG-PET mask (Ridgway et al., 2009). This mask was created using the SPM masking toolbox to produce an average binary mask, where the voxels from which to determine the 18FFDG metabolism parameter estimates were restricted to an explicit mask. The latter resulted from optimal thresholding of voxels in each image based on their correlation with an average image (i.e., the average of 18FFDG-PET images from the CG scans) with voxels not meeting the optimality criterion set to zero (masked) (Perani et al., 2014). This mask was applied (i.e., explicit masking option in SPM8-GLM models) to restrict subsequent single-subject statistical analyses only to within-brain voxels in order to eliminate variance due to inter-subject variation and noise from outside the brain. The SPM comparison between each single 18FFDG-PET scan and the healthy control group of scans essentially provides regional differences in relative glucose metabolism by means of a t-statistic for each voxel (SPM-t maps). Clusters of decreased metabolism were considered significant when they met a significance level of *p* = 0.05, corrected for multiple comparisons with the family-wise error (FWE) option at the voxel level, and contained more than 100 voxels (Perani et al., 2014).

## Results

### Demographic and clinical data

Three Hundred and fifty consecutive patients with mixed cognitive-depressive symptoms were screened between January 2010 and December 2014. Among these patients, 94 were excluded since 34 were diagnosed as affected by FTLD, 9 were affected by parkinsonism, 2 had a brain mass at MRI, 7 had abnormal cell count at CSF analysis, 35 showed lacunar infarcts at brain MRI, 4 had subclinical epileptic seizures, and 3 showed hydrocephalus.

**Figure 1:**
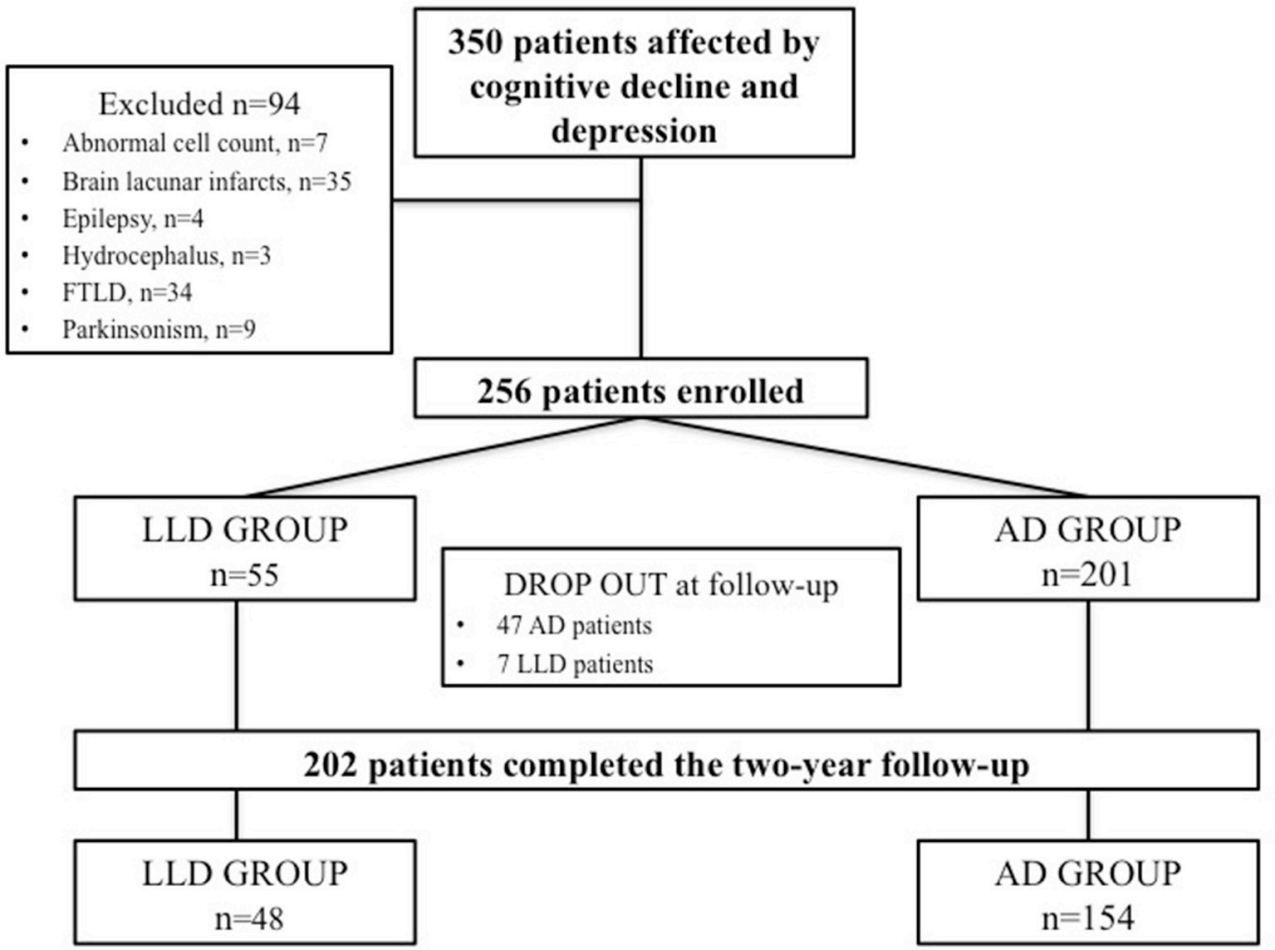
Flow-chart of the study.

Therefore, 256 patients were diagnosed as affected by cognitive decline and depression and entered the study. On the basis of the criteria for AD diagnosis, 201 participants were included in the AD group. Conversely, 55 patients showing the diagnostic criteria for LLD constituted the LLD group. Forty-seven patients included in the AD group and 7 patients included in the LLD group discontinued the follow-up because of poor compliance. Finally, 154 AD patients and 48 LLD patients completed the 2-year follow-up and were considered for the analysis. Flow-chart of patients' recruitment is depicted in Figure 1. The control group consisted of 48 subjects age- and sex-matched with AD and LLD groups. Demographic and clinical features of patients and controls are summarized in Table 1.

**Table 1:** Demographic and CSF data obtained at baseline of AD and LLD patients and controls.

	**LLD (*****n*** = **48) (mean** ±***SD*****)**		**AD patients (*****n*** = **154) (mean** ±***SD*****)**		**Controls (*n* = 58) (mean ±*SD*)**
Age (years)	70.98 ± 3.26		71.99 ± 4.01		67.89 ± 4.95
Sex	19M 29F		71M 83F		28M 30F
T-tau (pg/mL)	205.42 ± 83.21^*^		676.59 ± 373.53^◦^		252.89 ± 43.26
P-tau (pg/mL)	33.46 ± 8.56^*^		86.67 ± 51.28^◦^		32.75 ± 5.21
Aβ_42_ (pg/mL)	837.33 ± 194.96^*^		306.33 ± 105.69^◦^		921.96 ± 69.55
	**Baseline**	**Follow-up**	**Baseline**	**Follow-up**	
MMSE	20.91 ± 3.27^#^	24.35 ± 2.75^*^	20.20 ± 2.84^◦^	17.06 ± 3.28	29.08 ± 0.90
PHQ-9	17.58 ± 2.21	8.75 ± 1.83^*^	17.01 ± 2.41	13.5 ± 3.34	NA

*MMSE and PHQ-9 data of AD and LLD patients obtained at baseline and follow-up*.

* LLD vs. AD, p < 0.001;

◦ AD vs. Controls, p < 0.001;

# *LLD vs. Controls, p < 0.001*.

*F, female; M, male; MMSE; Mini Mental State Examination; PHQ-9, Patient Health Questionnaire 9; T-tau, total tau proteins; P-tau, phosphorylated tau proteins; Aβ_42_, beta amyloid 1-42; NA, not applicable*.

### MMSE and PHQ-9 data

At the time of diagnostic workup, all patients underwent MMSE and PHQ-9 assessments. Once patients were divided in two groups on the basis of current diagnostic criteria for AD and LLD, we found no significant difference comparing MMSE (20.91 ± 3.27 vs. 20.20 ± 2.84, Table 1) and PHQ-9 (17.58 ± 2.21 vs. 17.01 ± 2.42, Table 1) between LLD and AD groups. At the end of the 2 year-follow-up, AD patients showed significantly lower MMSE scores compared to LLD patients (17.06 ± 3.28 vs. 24.35 ± 2.75, *p* < 0.01, Table 1). Conversely, LLD patients presented the significant reduction of PHQ-9 total score compared to AD patients (8.75 ± 1.83 vs. 13.50 ± 3.34, *p* < 0.01, Table 1).

### CSF data

**Figure 2:**
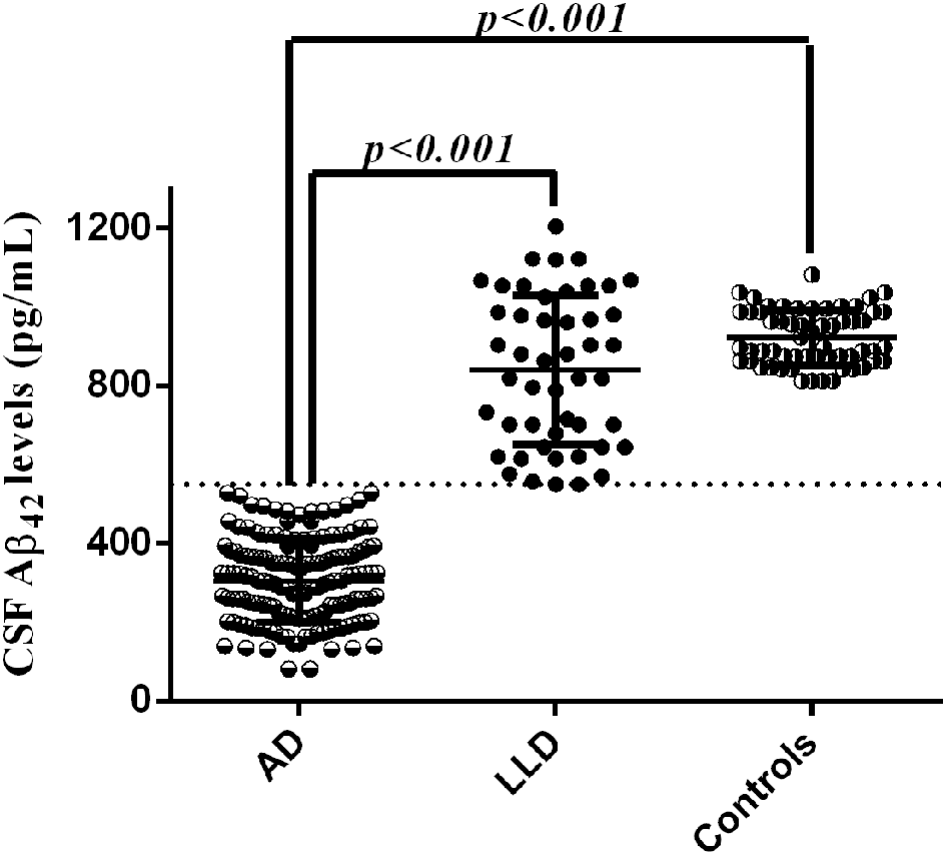
CSF Aβ_42_ levels in LLD, AD, and control groups. Cut-off of pathological CSF Aβ_42_ levels was set at 500 pg/mL.

LP was performed at baseline during the diagnostic work-up in all patients. We found that LLD patients showed significant higher Aβ_42_ CSF levels (837.33 ± 194.96 vs. 306.33 ± 105.69 pg/mL, *p* < 0.001) coupled with significant lower t-tau (205.41 ± 83.22 vs. 676.60 ± 373.53 pg/mL, *p* < 0.001) and p-tau (33.46 ± 9.56 vs. 86.67 ± 51.28 pg/mL, *p* < 0.001) CSF levels compared to AD patients. We also found that Aβ_42_ CSF levels of LLD patients (range between 550 and 1204 pg/mL) did not overlap with those of AD patients (range between 82 and 528 pg/mL) (Figure 2). Moreover, LLD patients showed no differences in the Aβ_42_ (813.33 ± 232.96 vs. 921.96 ± 69.55), t-tau (205.41 ± 83.22 vs. 209.48 ± 87.63), and p-tau (33.46 ± 9.56 vs. 39.62 ± 12.22) CSF concentrations compared to controls. At contrast, in AD patients we documented the significant decrease of Aβ_42_ CSF levels and the significant increase of CSF t-tau and p-tau levels compared to Control group (Table 1). Finally, all the AD patients but no LLD patients showed the t-tau/abeta ratio ( > 0.52) consistent with AD pathology.

### 18FFDG-PET analyses

**Figure 3:**
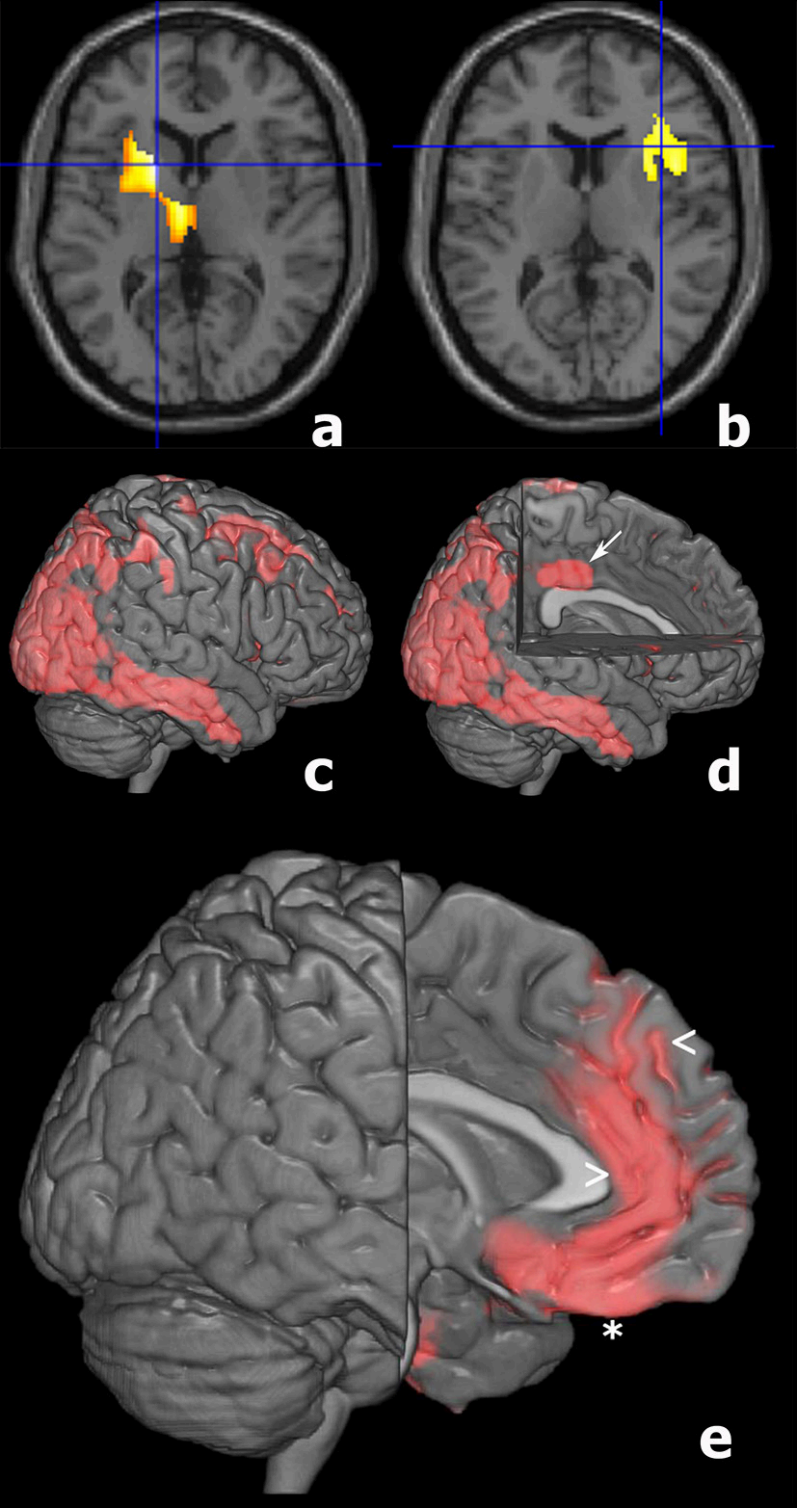
T1 magnetic resonance imaging superimposition of SPM results of a subgroup of LLD patients compared to Control group showing in: (patient 1, **a**) the reduced glucose consumption in the left thalamus and putamen (P Fwe corr = 0.001; P FDT corr = 0.001; Z score = 5.89; cluster extent = 1002); (patient 2, **b**) a reduced metabolism in the right insula (P Fwecorr = 0.006; P FDT corr = 0.013; Z score = 4.41; cluster extent = 485); (patient 3, **c,d**) 3D brain rendering showing the results of SPM analyses showing a significant hypometabolism in right parietal and temporal cortex (**c**, red) and in the left limbic cortex (**d**, arrow); (patient 4, **e**) 3D brain rendering showing the results of SPM analyses documenting a significant hypometabolism in left frontal cortex (red), and particularly in left rectal gyrus (^*^), medial frontal gyrus (<), and anterior cingulate cortex (>).

SPM analyses showed significant differences between AD and LLD groups in 18FFDG-PET assessment performed at the time of the diagnostic work-up. We found the reduction of 18FFDG-PET uptake in bilateral temporal and parietal cortices in AD than LLD patients (Table 2). At contrast, we did not find differences in cerebral glucose metabolism between LLD and Control groups. However, when the analysis was performed at an individual level between each LLD patient and the whole control group, we found that LLD patients were affected by significant cerebral glucose hypometabolisms in different brain cortical and subcortical areas. In fact, single subject SPM analyses showed heterogeneous patterns of 18FFDG hypometabolism in a subgroup of LLD patients (19/48). In particular, we documented cerebral glucose hypometabolism in putaminal and thalamic nuclei (Figure 3, patient LLD-a); reduction of brain glucose consumption in the insula (see Figure 3, patient LLD-b); glucose hypometabolism in right parietal and temporal cortex (see Figure 3, patient LLD-c) and in left limbic cortex (see Figure 3, patient LLD-d); glucose hypometabolism in left frontal cortex, and particularly in left rectal gyrus, medial frontal gyrus, and anterior cingulate cortex (see Figure 3, patient LLD-e).

**Table 2:** Statistical parametric mapping comparisons of 18F-FDG uptake between LLD and AD groups.

**Analysis**	**Cluster level**					**Voxel level**		
**LLD—AD**	**Cluster p(FWE-corr)**	**Cluster p(FDR-corr)**	**Cluster extent**	**Cortical Region**	**Z score of maximum**	**Talairach coordinates**	**Cortical region**	**BA**
	0.004	0.000	16427	R Parietal	5.19	2, −48, 34	Precuneus	7
				R Parietal	5.04	52, −50, 46	Inferior parietal lobule	40
				R Temporal	5.00	62, −34, −2	Middle temporal gyrus	21
	0.014	0.000	13149	L Parietal	5.35	0, −48, 34	Precuneus	31
				L Temporal	5.17	−54, −28, −0	Middle temporal gyrus	21
				L Temporal	5.05	−52, −54, −14	Inferior temporal gyrus	20

*FDG-PET was obtained at baseline in LLD and AD patients*.

*In the “cluster level” section on left, the number of voxels, the corrected P-value of significance and the cortical region where the voxel is found, are all reported for each significant cluster. In the “voxel level” section, all of the coordinates of the correlation sites (with the Z-score of the maximum correlation point), the corresponding cortical region and BA are reported for each significant cluster. L, left; R, right; BA, Brodmann's area. In the case that the maximum correlation is achieved outside the gray matter, the nearest gray matter (within a range of 5 mm) is indicated with the corresponding BA*.

## Discussion

Consisting evidence indicates that cognitive impairment and depression frequently coexist in the elderly (Kobayashi and Kato, 2011; Richard et al., 2013). Accordingly, AD and LLD are two different neuropsychiatric disorders possibly sharing the same clinical picture of cognitive decline and depression (Fischer, 1996; American Psychiatric Association, 2000; McKhann et al., 2011). In particular, the early differential diagnosis between AD and LLD may be challenging since patients suffering from LLD may be misdiagnosed as affected by AD, and viceversa (Richard et al., 2013; Morimoto et al., 2015). Accordingly, identifying the pathological process underlying this mixed cognitive-depressive clinical picture in the elderly represents a central point for setting the proper treatment strategy and appropriate clinical follow-up. Hence, currenct diagnostic criteria have been provided for AD in order to ensure the correct diagnosis and clinical-follow-up. They include β-amyloid and/or PET analysis to confirm the amyloidaphy at the basis of the dementing AD process. (McKhann et al., 2011).

Here, we present the data of a 2-year follow-up study conducted in a large population of elderly patients manifesting both cognitive deterioration and depression, who performed a complete diagnostic workup at baseline, including CSF and brain 18FFDG-PET assessments.

Briefly, in this study we considered patients affected by depression and dementia who performed LP and FDG-PET analysis at baseline and then distributed in two groups based on the current diagnostic guidelines for AD and LLD (American Psychiatric Association, 2000; McKhann et al., 2011). Patients with a biomarkers profile consistent with AD pathology were included in the AD group, (McKhann et al., 2011) whereas the remaining patients, showing depressive symptoms with dementia, but without AD biomarkers alteration, constituted the LLD group (American Psychiatric Association, 2000). At the end of the 2-year follow-up, in order to test the diagnosis performed at baseline thanks to the CSF and brain 18FFDG-PET analysis we compared the baseline data between AD and LLD groups. Moreover, both groups were compared to a control group constituted by not demented and depressed elderly. As expected, we documented higher CSF tau proteins levels and lower CSF Aβ_42_ concentrations in the AD group than both LLD and controls. Moreover, AD patients showed the bilateral temporal and parietal glucose hypometabolism with respect to LLD and controls. Notably, CSF biomarkers and brain 18FFDG-PET did not differ between LLD and controls.

The clinical follow-up permitted the longitudinal analysis of neuropsychological test, which documented the significant improvement of depression and cognitive performances in patients included at baseline in the LLD group. We also observed the significant worsening of cognition associated with the irrelevant change of depression in patients included in the AD group.

Consistently, if we consider the CSF AD biomarkers analysis performed at baseline, all patients showing depression and dementia with a preserved CSF profile included in the LLD group at baseline did not worsen or even improved their cognitive status once their depressive symptoms resolved; whereas patients showing biomarkers consistent with AD pathology and included in the AD group progressively deteriorate their cognition with unsignificant changes of depression.

Hence, these finsings point out that in patients affected by depression and dementia the CSF AD biomarkers analysis may offer a concrete support in early differentiating LLD from AD.

As well-documented, the CSF profile of AD patients shows decreased Aβ_42_ and increased t-tau and p-tau CSF levels, reflecting the neurodegenerative processes underling the disease, which is typically featured by extracellular β-amyloid plaques deposition and NFTs pathology (McKhann et al., 2011). CSF p-tau levels have been identified as a promising diagnostic test for AD (Mitchell and Brindle, 2003). Accordingly, tau proteins are the main component of paired helical filaments (PHFs) forming the NFTs (Grundke-Iqbal et al., 1986; Lee et al., 1991). Tau proteins in PHFs and NFTs are abnormally hyperphosphorylated and are widely present in the brain of AD patients. In agreement with this evidence, our population of AD patients showed higher CSF t-tau and p-tau levels compared to both LLD and controls. Conversely, CSF t-tau and p-tau levels did not differ between LLD patients and controls. However, considering the three AD CSF biomarkers (t-tau, p-tau, and Aβ_42_) we found that exclusively the individual values of CSF Aβ_42_ levels did not overlap between LLD and AD patients, since all the LLD patients showed CSF Aβ_42_ levels above the cut-off value for AD diagnosis (≥500 pg/mL). Although CSF Aβ_42_ levels were above the cut-off in LLD patients, they appeared dispersed when compared to those of controls (see Figure 2). This finding seems not to have a biological sense and needs further analysis, since the pathological mechanisms at the basis of LLD are being actually under investigation. However, all LLD patients also showed the tau/Aβ_42_ ratio under the established cut-off for AD diagnosis ( > 0.52) (Liguori et al., 2017). Therefore, our present CSF study highlights that cognitive deficits complained by LLD patients were unrelated to AD-like neurodegeneration, by reason of LLD patients showed no pathological changes in Aβ_42_ and tau proteins contents, but a CSF biomarker profile similar to non-demented controls (Lee et al., 1991). These data are consistent with previous reports showing that brain tissues from LLD patients did not show the pathological hallmark of AD pathology (Lee et al., 1991; Wilson et al., 2016). Therefore, amyloidopathy confirmed by CSF or PET analysis is the core criteria for AD diagnosis, as stated by the National Institute on Aging-Alzheimer's Association workgroups (McKhann et al., 2011). In keeping with the criteria already suggested, we confirmed that the CSF biomarkers of amyloidopathy coupled with the clinical criteria of dementia significantly support the pathophysiological process of AD at the basis of the neurodegenerative process.

It is known that depression is the most frequent neuropsychiatric symptom associated with AD, which can even precede the underlying neurodegenerative process (Marin et al., 1997; Purandare et al., 2001; Rushing et al., 2014). Moreover, depression has been considered either a risk factor or a prodromal condition of AD, as confirmed by previous CSF data documenting that depressed, but cognitive intact, patients show a CSF profile consistent with preclinical AD (Buerger et al., 2003; Pomara et al., 2012, 2016). Nevertheless, patients with LLD may appear demented because of the severity of their depressive symptoms, independently of the increased risk for developing dementia (Elderkin-Thompson et al., 2003; Beblo et al., 2011).

In this study, we did not evaluate LLD as a prodromal symptom for AD pathology, but we were focused on patients affected by LLD with associated dementia. Moreover, in agreement with the aim and methodology of the study, the control group was selected following the inclusion/exclusion criteria and did not show pathological CSF AD biomarkers indicative of preclinical AD.

Considering the complex interplay between dementia and depression, the co-occurrence of these symptoms in the elderly may complicate the differential diagnosis between LLD and AD, thus possibly precluding the appropriate pharmacological treatment and clinical follow-up. However, a diagnostic set designed at discriminating whether clinical symptoms are due to LLD or AD is not yet defined. In keeping with this observation, the neuropsychological, clinical, and neuroimaging instruments currently proposed to early differentiate LLD from AD remain challenging and to a certain extent debatable. Previous clinical studies tried to differentiate LLD from AD using neuropsychological tools; however, since LLD and AD patients may share the same cognitive deficits, including memory impairment, executive dysfunction, and slow processing speed, a general agreement about an univocal neuropsychological profile for LLD has not been achieved (Butters et al., 2000; Hohman et al., 2011; Rushing et al., 2014; Callahan et al., 2016). Thus, neuropsychological test may be considered sensitive but not specific for LLD diagnosis (Elderkin-Thompson et al., 2003; Beblo et al., 2011).

Likewise, also neuroimaging may not distinguish LLD from AD, as either brain MRI or 18FFDG-PET can variously document brain atrophy, cerebral glucose hypometabolism, and reduced blood flow in both conditions (Kumar et al., 1993; Alexopoulos, 2002; Cho et al., 2002; Herholz, 2003; Su et al., 2006; Sexton et al., 2013; Du et al., 2014; Sivakumar et al., 2015). In fact, although atrophy and glucose hypometabolism in bilateral temporal and parietal regions characterize AD, functional and structural brain pathological changes may also occur in LLD (Kumar et al., 1993; Alexopoulos, 2002; Cho et al., 2002; Herholz, 2003; Su et al., 2006; Sexton et al., 2013; Du et al., 2014; Sivakumar et al., 2015). Functional studies in LLD patients showed glucose hypometabolism in frontal and temporal areas, as well as in caudate and thalamus, and a decreased cerebral blood flow in temporal and parietal regions (Kumar et al., 1993; Alexopoulos, 2002; Cho et al., 2002; Herholz, 2003; Su et al., 2006; Sexton et al., 2013; Du et al., 2014; Sivakumar et al., 2015). Brain structural studies in LLD evidenced gray matter volume alterations in multiple fronto-striatal-temporal regions, such as hyppocampus, cingulated cortex, and putamen (Kumar et al., 1993; Alexopoulos, 2002; Cho et al., 2002; Herholz, 2003; Su et al., 2006; Sexton et al., 2013; Du et al., 2014; Sivakumar et al., 2015). Here we documented that, unlike AD patients showing a homogeneous pattern of temporal and parietal 18FFDG-PET hypometabolism, a sub-set of LLD patients displayed 18FDG hypometabolism involving several cortical and subcortical brain areas (see Figure 3). Moreover, although we found no significant differences in cerebral glucose metabolism between control and LLD groups, the single subject analysis of 18FFDG metabolism between each LLD patient and the control group documented individual significant differences in several brain areas, reflecting the functional impairment of different cortico-subcortical brain regions (see Figure 3). Therefore, we confirmed the occurrence of heterogeneous patterns of glucose hypometabolism at 18FFDG-PET in LLD, but we also suggested that it could be imprudent or inaccurate to distinguish LLD from AD patients on the basis of neuroimaging findings, since the extreme variability of functional patterns, which may overlap between LLD and AD.

In conclusion, in the context of the major public health concern caused by AD, we renovate the proposal to perform all the CSF AD biomarkers analysis in elderly patients with depression and dementia in order to reduce possible diagnostic inaccuracies between AD and LLD (Olsson et al., 2016; Herukka et al., 2017; Simonsen et al., 2017).

## Author contributions

CL: study concept, acquisition of data, data analysis and interpretation, statistical analysis, drafting the manuscript; AC: PET data analysis, statistical analysis; GS: conception and design of the work study supervision, critical revision of the manuscript for important intellectual content; FF: acquisition of data; GMS: CSF data acquisition and analysis and manuscript draft; NM: study supervision; OS: critical revision of the manuscript for important intellectual content; MP: supervision, data analysis and interpretation, statistical analysis, drafting the manuscript.

### Conflict of interest statement

The authors declare that the research was conducted in the absence of any commercial or financial relationships that could be construed as a potential conflict of interest. The reviewer AD and handling Editor declared their shared affiliation.
